# Musician’s dystonia: a perspective on the strongest evidence towards new prevention and mitigation treatments

**DOI:** 10.3389/fnetp.2024.1508592

**Published:** 2025-01-22

**Authors:** Joy Grifoni, Valeria Crispiatico, Anna Castagna, Rosa Maria Converti, Marina Ramella, Angelo Quartarone, Teresa L’Abbate, Karolina Armonaite, Luca Paulon, Francescaroberta Panuccio, Franca Tecchio

**Affiliations:** ^1^ Faculty of Psychology and of Engineering, Uninettuno University, Rome, Italy; ^2^ Laboratory of Electrophysiology for Translational neuroScience LET’S, Institute of Cognitive Sciences and Technologies ISTC, Consiglio Nazionale delle Ricerche CNR, Roma, Italy; ^3^ Department of Psychology, University of Milano-Bicocca, Milan, Italy; ^4^ IRCCS Fondazione Don Carlo Gnocchi, Milan, Italy; ^5^ IRCCS Centro Neurolesi “Bonino Pulejo”, Messina, Italy; ^6^ Engineer Freelance, Rome, Italy; ^7^ Department of Human Neurosciences, Sapienza University of Rome, Rome, Italy

**Keywords:** task-specific focal dystonia, sensory-motor integration, psychic trauma, multi-sensory multimodal rehabilitation, feedback synchrony plasticity: the FeeSyCy principle governing networks, network physiology

## Abstract

This perspective article addresses the critical and up-to-date problem of task-specific musician’s dystonia (MD) from both theoretical and practical perspectives. Theoretically, MD is explored as a result of impaired sensorimotor interplay across different brain circuits, supported by the most frequently cited scientific evidence—each referenced dozens of times in Scopus. Practically, MD is a significant issue as it occurs over 60 times more frequently in musicians compared to other professions, underscoring the influence of individual training as well as environmental, social, and emotional factors. To address these challenges, we propose a novel application of the FeeSyCy principle (feedback-synchrony-plasticity), which emphasizes the pivotal role of feedback in guiding inter-neuronal synchronization and plasticity—the foundation of learning and memory. This model integrates with established literature to form a comprehensive framework for understanding MD as an impaired FeeSyCy-mediated relationship between the individual and their environment, ultimately leading to trauma. The proposed approach provides significant advantages by enabling the development of innovative therapeutic and preventive strategies. Specifically, it lays the groundwork for multimodal psycho-physical therapies aimed at restoring balance in the neural circuits affected by MD. These strategies include personalized psychotherapy combined with physical rehabilitation to address both the psychological and physiological dimensions of MD. This integration offers a practical and value-added solution to this pressing problem, with potential for broad applicability across similar conditions.

## 1 Introduction

Task-specific dystonia is a movement disorder characterized by a usually painless loss of dexterity specific to a particular motor skill (Sadnicka et al., 2018). In musicians, the disorder emerges as loss of finger motor coordination or embouchure exclusively when playing the musical instrument (Candia et al., 2003), giving rise to hand or embouchure musician’s dystonia (MD; Stahl and Frucht, 2017). MD of the hand affects musicians who play strings and plucked strings such as violinists, cellists, guitarists, pianists and more rarely wind and brass players. MD of the embouchure affects brass and woodwind players, involving the perioral, lingual, and facial musculature (Frucht et al., 2001).

The causes of MD are still not yet completely clarified, but it is believed to arise from a combination of genetic predisposition and environmental and psychic factors, affecting the brain’s motor control system. As for the genetic hypothesis, it is partly supported by evidence that up to 25% of patients with MD have another affected family member with dystonia. Furthermore, a recent genome analysis found an association with the arylsulfatase G gene (ARSG) in both musician’s hand dystonia and writer’s cramp, but a specific causal mutation within this gene has not yet been identified (Stahl and Frucht, 2017; Nibbeling et al., 2015). Possible predisposing risk factors for MD include a positive family history of dystonia, a history of musculoskeletal injury, nerve entrapment or overuse syndrome, and obsessive personality traits (Rozanski et al., 2015).

MD is the most common form of focal task-specific dystonia, with a prevalence of 1:100 compared to 1:6,600 for idiopathic dystonia (Rozanski et al., 2015). The prevalence of MD varies depending on the instrument played, with musicians playing piano, violin, guitar, and brass instruments being about 85% (Aránguiz et al., 2015), and embouchure MD accounting for 13%–14% of MD (Butler and Rosenkranz, 2006; Jabusch and Altenmüller, 2006), and cervical dystonia involving 1%–2% of musicians with MD.

Advancements require sharing standardized measures among experts in the field to evaluate the severity of MD symptoms, for diagnosis and assessing the success of treatments. As key reference we ground on the work of Peterson and collegues (Peterson et al., 2013), which developed a wide overview of the rating scales used in MD. The vast majority of studies use subjective-reported or clinician-reported scales, while objective scales started to be introduced (see Table 1). To date, the Arm Dystonia Disability Scale (ADDS) and Tubiana and Chamagne Scale (TCS) appear to be the most used tools for evaluating dystonia severity, with the TCS being the most specific for MD (Catellani et al., 2024).

**TABLE 1:** Available scales to score MD severity.

Scale name	Description	Ref
Clinician-reported scales		
Arm Dystonia Disability Scale (ADDS)	An ordinal scale scoring motor function impairment in 7 different manual activities (one of which is playing an instrument)	Fahn (1989)
Tubiana and Chamagne Scale (TCS)	A Likert-type scale assessing musical capabilities	Tubiana and Chamagne (1993)
Frequency of Abnormal Moements scale (FAM)	A video-based scale scoring abnormal digit movements (rate or % of time)	Spector and Brandfonbrener (2005)
Subjective-reported scales		
Visual Analog Scale (VAS)	Different versions of ordinal scale assessing performance improvements during short symptom-evoking passages or patient’s perception of impairment	Rosenkranz et al. (2009) Cole et al. (1995)
Dystonia Evaluation Scale (DES)	Ordinal scale rating performance during movement exercises and symptom-evoking pieces	Candia et al. (1999) Candia et al. (2002)
Technical Ability and Performing Scale (TAPS)	Scale assessing the subjective perception of MD burden on musical performance in clinical setting	Ramella et al., 2022
Objective scales		
Kinematics	Instrumental assessment of the position and rotations of digit segments over time, evaluating velocity and acceleration	Candia et al. (1999) Candia et al. (2002)
Musical Instrument Digital Interface (MIDI)–based Scale Analysis	A MIDI interface records velocity and timing during different musical performances	Jabusch and Altenmüller (2004)

The present Perspective integrates the most robust evidence on MD pathophysiology and interventions (Section 2) with our model of neuronal network governing principles (Section 3), highlighting its alteration in MD (Section 4). This foundation supports the development of novel therapeutic strategies, which are detailed in Section 5.

## 2 Current treatments

Among approaches tested over the years to address MD, the most common is botulinum neurotoxin (BoNT) injections (Cohen et al., 1989; Jankovic and Schwartz, 1990; Kuru et al., 2010; Gupta and Pandey, 2021). This commonly used treatment is effective for about 12–16 weeks with the effect dose-dependent. Cautiousness is indicated, as BoNT is operator dependent; it depends on guides (electromyography/ultrasound), doses, and expertise. It is not possible to consider BoNT as a recommended treatment, but some good results are published in finger flexors dystonia (Gupta and Pandey, 2021). However, in the case of embouchure dystonia, most authors report a worsening of musical performance with the use of botulinum toxin (Hallett, 2018; Weise et al., 2019; Lee et al., 2021). Furthermore, a recent investigation (Ioannou et al., 2023) revealed that, compared to the control group, the thickness and force strength of injected flexor digitorum superficialis (FDS) and profundus (FDP) muscles were decreased by about 10%–12% with respect to the non-injected body side, with the extent of weakness and atrophy significantly predicted by the total amount of BoNT injected during the entire treatment period and the time since the last injection not predicting the amount of strength and muscle mass recovery after cessation of treatment.

Non-pharmacological approaches were developed mainly involving various neuromuscular programs (Jabusch and Altenmüller, 2004; Enke and Poskey, 2018). While recent reviews observe that most studies were applied on a very small sample of musicians with MD, with follow-up assessment absent or very short, and the evaluations often based on subjective nominal or ordinal scale, employing study designs without any type of randomization to placebo or alternative treatments or blindness, we will report here only clear indications originating in focal task specific hand dystonia and implemented in musicians.

“Sensory tricks,” also called alleviating exercises, are known to provide temporary relief of dystonic symptoms (Kaji et al., 1995; Loyola et al., 2013; Dagostino et al., 2019), usually involving the alteration of tactile or proprioceptive feedback of the dystonic districts. In examples, musicians often experience a marked improvement in fine-motor control when they play with a latex glove or when holding an object, such as chewing gum, between their fingers, thus modifying the somatosensory input information (Zeuner et al., 2005). Usually, the effects are very rapid in changing the motor pattern, but they last for a limited time. Sensory motor retuning (SMR) treatment has been introduced in a seminal study in 2002 (Candia et al., 2002) introducing the immobilization with splints of one or more fingers other than the task-specific dystonic finger. The dystonic finger/s performed repetitive exercises in coordination with the non-splinted ones for 1–2 h per day for eight consecutive days under therapist supervision. The subjects were then asked to continue the practice for 1 h per day for 1 year. The splint maintains the fingers of the person with MD in their characteristic rest position on the instrument, simulating the positions experienced during normal performance. In this way, the dystonic finger can participate in the alternating movements of the individual fingers with all the possible permutations of the other fingers of the dystonic hand. In seminal studies, the effects of the treatments were assessed by dystonic finger dexterity quantified by movement speed and accuracy: for example, pre- and post-treatment segments of the movement slopes of a patient’s right D3 (RD3) and left D3 (LD3) were assessed while performing a trill-like task at a selected fast and free speed. Before treatment, the recorded movements of the dystonic finger (RD3) were irregular and uncontrolled compared to the movements of the homologous LD3, which served as control finger. These differences are no longer present after treatment.

Sakai (2006) developed a motor control retraining technique in MD pianists, named “slow-down exercise” (SDE). During the exercise program, patients undergo basic movement training at decreased speed, ensuring that the dystonic patterns do not occur at this reduced speed. The pianists would increase metronome speed every 2 weeks as long as they could maintain a normal movement pattern. Berque and colleagues (Berque et al., 2010) developed a standardized protocol combining SMR plus SDE intervention over a 12-month period.

## 3 Goal-dependent feedback-synchrony-plasticity principle that governs the interaction of the person with the environment (FeeSyCy)

To overcome the limitations of current therapeutic strategies, a deeper understanding of the mechanisms underlying MD is essential. Our approach begins with a model that helps us explore the relationship between individuals and the world they inhabit, integrating both electrophysiological processes and emotional and social mechanisms in a cohesive framework.

**FIGURE 1:**
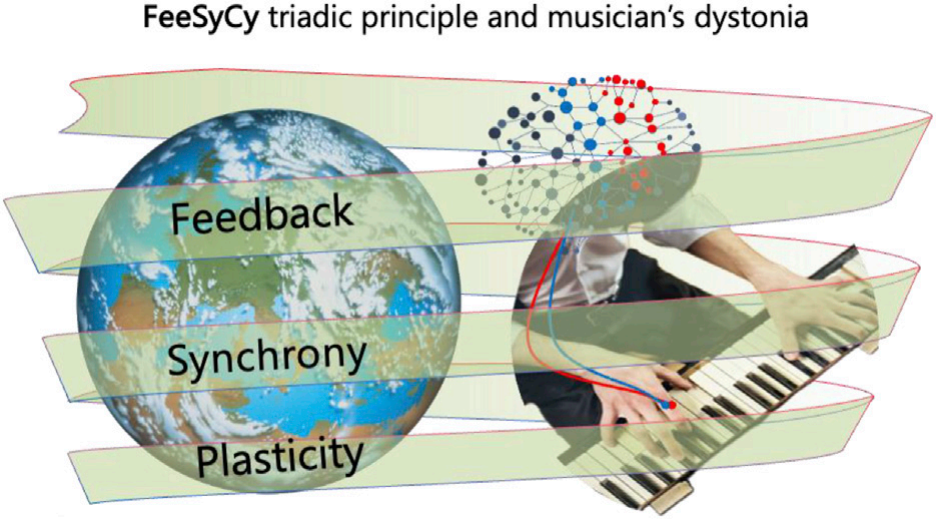
Neuronal network mediated alterations in musician’s dystonia. An outline of the FeeSyCy triadic principle—feedback, synchrony, plasticity—that governs the body-brain interaction with the environment and is involved in the altered sensorimotor integration seen in the musician’s dystonia.

Our description of the triadic principle of feedback, synchrony, and plasticity (FeeSyCy, Tecchio et al., 2020) (Figure 1) incorporates the dynamic nature of neuronal networks, which are driven by goal-dependent feedback loops. These loops give rise to the adaptive interaction between the body and brain with the environment, allowing goals to be identified and pursued through multiple interconnected processes within the individual.

The whole brain can be seen as an ensemble of neurons coordinating the body-brain network’s interaction with the environment. In this context, output (motor actions) depends on sensory input (via somatic, proprioceptive, visual, and auditory receptors): critically the physiology stands on input that, in turn, depends on the output (Tecchio et al., 2020; Persichilli et al., 2022). This reciprocal relationship is fundamental to the feedback loop that underlies adaptive behavior (Rossi et al., 1998). When motor actions are performed, sensory feedback from the environment is shaped by the brain in accordance with the desired goal, continually refining and adjusting the system to reduce the difference between top-down expectations and bottom-up sensory inputs (Fink et al., 2014).

Within this feedback loop, the brain’s neurons organize themselves through locally specific dynamic synchronizations among nodes of functional networks (Tecchio et al., 2008a). These synchronizations occur at the subsystem level, typically accompanied by desynchronizations with wider cortical regions, such as those giving rise to the reduction of occipital alpha power when opening the eyes. The modulation of synchrony leads to either the continued execution of planned actions or corrective adaptations, depending on the feedback received from the environment.

Central to this process is the *Hebbian learning rule*, which governs how neurons modify their output in response to synchronized inputs. When two input signals arrive simultaneously at a neuron, its likelihood of firing increases, thus facilitating message transmission within dynamic networks along the whole lifespan (Kerkman et al., 2022; Madadi Asl et al., 2022; Hill and Den Hartigh, 2023; Hristovski et al., 2023). This mechanism underlies key learning processes such as trial-and-error and imitation, enabling neurons to adapt their output based on the distance between the expected outcome and the current state. If this distance is small, behavioral adaptations occur within the existing network framework (working adaptation). Significant structural changes arise if the distance is large, reflecting new skill acquisition and plastic adaptation for learning. We use the term network plasticity, defined as a change of network output in response to the same input, referring to the brain’s capacity to adapt structurally and functionally, which is an ability manifested at multiple levels, including synaptic plasticity (through potentiation and depression phenomena) and neuronal structural modification, including pruning when the brain gets rid of neurons and connections that it no longer requires, and branching when new synaptic connections are made. These changes are further supported by modifications in myelin sheaths, which adjust the timing of information transmission with high precision. This multi-layered plasticity to ensure system adaptations critically requires not only active behavior but also sleep, when synaptic strengths are renormalized, maintaining high the potential for new acquisitions in the neural network (Papadakis et al., 2022; Rizzo et al., 2023).

In sum, the FeeSyCy triadic principle operates through a feedback loop in which sensory input and motor output continuously influence each other, transduced by neuronal synchronizations that drive plastic changes. These processes work together to support goal-directed behavior, via learning for the ongoing adaptation of the body-brain system to the environment. The FeeSyCy principle subtends physiological action in the environment, at multiple levels, from simply motoric, to social, emotional, and affective.

## 4 MD mechanisms

### 4.1 Neurophysiological counterpart

Impairment in sensorimotor integration, crucial in the development of task-specific dystonia, is evidenced by findings from animal studies, behavioral research, and clinical reports utilizing neurophysiological and neuroimaging techniques in humans (Quartarone et al., 2006; Breakefield et al., 2008; Lin and Hallett, 2009). A seminal animal study (Byl et al., 1997) demonstrated that repetitive finger compression led to degradation of hand representation and impaired motor control, while a less repetitive strategy preserved sensory integrity and motor function. From that moment, dystonia was constantly associated with the repetitiveness of motor patterns influencing somatosensory representation and motor execution. Following the SMR model by Candia and colleagues (Candia et al., 2002), treatment effects were studied on somatosensory hand representation. Pre-treatment, finger relations differed between affected and unaffected hands, but post-treatment, contralateral finger representations resembled those of the less affected side and were ordered more according to homuncular principles (Candia et al., 2003). These physiological changes correlate with behavioral outcomes (Candia et al., 2003). Per the FeeSyCy principle, we expect synchronization alterations in MD. Investigations employing transcranial magnetic stimulation (TMS) protocols revealed the absence of local inhibitory mechanisms during movement initiation and maintenance phases in individuals with task-specific hand dystonia, leading to disrupted cortical inhibition-excitation dynamics (Desrochers et al., 2019). This disturbance affects cortical surround inhibition critical for skill acquisition and relies on inter-hemispheric balances (Pagliara et al., 2023; Bertoli et al., 2023). Notably, patients with task-specific hand dystonia exhibit disrupted cortical surround inhibition (Hallett, 2011) even during motor preparation (Beck et al., 2008). Altered excitation and inhibition balances in local motor cortex circuits, reflecting disrupted basal ganglia input, were evidenced by reduced excitability of cortical inhibitory circuits in patients with task-specific dystonia (Ridding et al., 1995). While no differences were observed in spectral properties between patients and controls in primary motor or somatosensory hand representations, these areas demonstrated reduced functional coupling during movement, along with excessive synchronization in patients compared to controls in the ongoing gamma band around 40 Hz (Tecchio et al., 2008b; Melgari et al., 2013). Concerning plasticity (Madadi Asl et al., 2022; McGowan et al., 2014), utilizing established non-invasive models of plasticity in humans (Stefan et al., 2000), it has been observed that the motor system exhibits abnormal increases in cortico-spinal excitability and attenuated intracortical inhibitory reinforcement following central associative and peripheral stimulation (Quartarone et al., 2003). Moreover, evidence confirms alterations in long-term potentiation and depression in MD (Quartarone et al., 2006).

While we emphasized the relevance of MD in the present context, the FeeSyCy principle is broadly applicable. Its explanatory potential extends across all task-specific dystonias, which, unlike other movement disorders, are primarily characterized by dysfunctions rooted in altered sensorimotor integration rather than by degenerative processes or structural lesions affecting motor pathways.

### 4.2 Psycho-social counterpart

While the neurophysiological profile described above is typical of task-specific dystonia of the hand, in musicians, the occupational dimension decisively modifies the impact of the symptomatology (Rosenkranz et al., 2005) consistent with epidemiological data that indicate MD as the most incident task-specific dystonia (Ioannou et al., 2016). It is immediate to consider that for a musician whose center of professional life is making music, the body is even more immediately at the center of the relationship with the rest of the world. For musicians, health tends to acquire two key dimensions in a dynamic balance, one that concerns the parts of the body directly involved in playing the instrument (especially the hand), and another that focuses on maintaining the whole person’s health. These two dimensions must be integrated in daily attention and care, deepening the awareness that learning through experience, aimed at mutual help and communication, is the key to protecting and recovering the whole body’s wellbeing (Schoeb and Zosso, 2012).

In the context of the increasingly clear nature of the human being determined within the experience of lifelong learning or traumas, and their deep impact on personal identity, (Grifoni et al., 2023), where one of the three main dimensions that index the development of a population is the length of the course of study, the learning approaches for musicians become critical in the training years and all along life. In fact, musicians typically start their training at a young age, with the need to develop movements automated through extensive repetitions, gradually increasing complexity, with the need to balance stereotyped production and emotional participation to the story they like to tell. Given the psycho-emotional pressure intrinsic with the public engagement of a musician’s activity, affecting the respiratory, circulatory, sensorimotor, and cognitive systems (Ioannou et al., 2016), the organization of individual and orchestral training is a critical element determining future evolution managing challenges in adapting to the demands of their profession. Indeed, trauma can have far-reaching consequences, extending beyond psychological distress to impact sensorimotor functionality in musicians. These repercussions are evident in the disruption of motor coordination, manifested as hesitations, involuntary movements, or complete motor blocks, which impede the expression of artistic intentions.

Furthermore, trauma-induced negations can exert influence on cognitive processes, including memory, attention, and decision-making, exacerbating the challenges faced by musicians during performance. Such cognitive impairments may lead to errors or inconsistencies in musical execution, undermining the quality of their performances. In light of these challenges, addressing trauma and its associated negations is paramount for preserving both physical and mental wellbeing in musicians. By acknowledging and confronting these negations, musicians can embark on a psychotherapeutic path towards reclaiming agency over their bodies and artistic expression. This process fosters resilience and facilitates a more comprehensive approach to musical performance and self-care for MD.

## 5 Discussion

We have reported on the most stable achievements on Musician’s Dystonia (MD). Modern rehabilitation approaches aim to restore a physiological position that supports musicians’ natural gestures, primarily through botulinum neurotoxin injections, sensory-motor retuning, and its variants. These strategies emphasize body awareness and the adjustment of positions for relaxation within a dynamic training program. The consolidated neurophysiological knowledge on the alteration of sensorimotor integration in MD, which coherently with the FeeSyCy *feedback-synchrony-plasticity* functioning principle that governs neuronal networks, is accompanied by impairments of intra-cortical synchronizations and plasticity, has recently been complemented by the growing awareness of the impact on symptoms of socio-emotional-cognitive experience.

In response, new therapeutic strategies are being developed to alleviate symptoms that profoundly affect musicians’ quality of life and often lead to career disruption. These strategies adopt a multidisciplinary approach involving neurology (Pérez and Preciado, 2019), physiotherapy, occupational therapy, and psychology (O’Neill, 2012; Zhang et al., 2020), integrated into personalized precision treatments. Within this integrated framework, the psychological protocol Eye Movement Desensitization and Reprocessing (EMDR) emerges as a comprehensive therapeutic approach to address the emotional trauma underlying MD. Notably, an enhanced method, referred to as EMDR+ (Grifoni et al., 2024), is proposed employing bi-modal visual (Stamatis et al., 2023) and auditory intervention of bilateral alternate stimulation, along with the inclusion of music in trauma-evocative and rewarding phases. EMDR + offers a unique potential for enriching therapeutic outcomes for musicians experiencing performance anxiety (O’Connor et al., 2021).

We believe that the proposed approach holds significant relevance to the field of Network Physiology, which investigates how physiological systems and subsystems interact, synchronize their functions, and integrate into networks to generate diverse physiological states and conditions in both health and disease. The FeeSyCy principle, emerging as a pivotal element in network functioning across multiple scales—ranging from small neuronal groups to the entire brain-body system and even society as a network of individuals—highlights the transformative potential of understanding systems as networks. This perspective opens the door to profoundly novel and effective interventions for symptom relief, such as in MD, and underscores the importance of incorporating this knowledge into teaching and training programs across various disciplines to prevent dysfunction and promote resilience.
